# Plasma Brain‐Derived Neurotrophic Factor (BDNF) and serum cortisol levels in a sample of workers exposed to occupational stress and suffering from Adjustment Disorders

**DOI:** 10.1002/brb3.1298

**Published:** 2019-06-14

**Authors:** Rodolfo Buselli, Antonello Veltri, Sigrid Baldanzi, Riccardo Marino, Alessandra Bonotti, Martina Chiumiento, Michelle Girardi, Luca Pellegrini, Giovanni Guglielmi, Liliana Dell'Osso, Alfonso Cristaudo

**Affiliations:** ^1^ Occupational Health Department, U.O. Medicina Preventiva del Lavoro Azienda Ospedaliero‐Universitaria Pisana Pisa Italy; ^2^ Unità Operativa Complessa Psichiatria di Pisa, Dipartimento della Salute Mentale e Dipendenze Azienda USL Toscana Nord Ovest Pisa Italy; ^3^ Fondazione BRF Onlus—Institute for Research in Psychiatry and Neuroscience Lucca Italy; ^4^ Section of Psychiatry, Department of Clinical and Experimental Medicine University of Pisa Pisa Italy; ^5^ Department of Translational Research and New Technologies in Medicine and Surgery University of Pisa Pisa Italy

**Keywords:** Adjustment Disorders, Brain‐Derived Neurotrophic Factor, cortisol, neuroplasticity, occupational medicine, work‐related stress

## Abstract

**Introduction:**

Decreased plasma BDNF (pBDNF) levels have been proposed as a biomarker in illness phases of mood disorders. Serum cortisol (seC) levels are an index of energy mobilization and stress. The aim of this cross‐sectional study was to evaluate pBDNF and seC levels in workers exposed to occupational stress and suffering from Adjustment Disorders (AD) compared to healthy workers.

**Methods:**

Plasma BDNF and seC levels were measured by means of specific immunoassays in 64 AD patients and 38 healthy controls. Perceived and occupational stress was evaluated in patients and controls using the Psychological Stress Measure (PSM) and the Job Content Questionnaire (JCQ). Psychopatological symptoms in patients were assessed using specific rating scales.

**Results:**

Plasma BDNF levels resulted significantly higher in patients than in controls, whereas no significant differences were found for seC levels. In patients but not in controls pBDNF levels showed a significant positive correlation with seC levels. Perceived stress levels were positively correlated with all psychopatological rating scales scores.

**Conclusions:**

BDNF could play a key role in the pathophysiology of stress‐related disorders and its peripheral levels elevation could contribute to protect neurons under stress. Further research is needed focusing on biomarkers for stress‐related disorders as a potential tool for the diagnosis and prevention of occupational diseases.

## 1 BACKGROUND

Work‐related stress is an emerging risk in occupational medicine, with deep impact on workers' performance and global health (Wang et al., 2014). The exposure to chronic work‐related stress could lead to psychiatric occupational diseases. In Italy, the recent list of occupational diseases with mandatory reporting contains the group of “Mental and psychosomatic disorders related to work organization dysfunction” which includes Post‐Traumatic Stress Disorder (PTSD) and Adjustment Disorders (AD) (INAIL, 2003).

Adjustment Disorders occur within 3 months after one or more objectively identified stressful events, and do not persist for more than additional 6 months once the stressor or its consequences terminated. They are characterized by heterogeneous emotional or behavioural symptoms belonging to the anxiety area or to the depressive one, which cause a significant impairment in social, occupational, or other important areas of functioning (American Psychiatric Association, 2013). The last version of the Diagnostic and Statistical Manual of Mental Disorders (DSM‐5) places AD along with PTSD in the “Trauma‐ and Stressor‐Related Disorders” section. The differential diagnosis between depressive episodes or anxiety disorders, above all if mild/moderate, and AD is often not simple even if it is crucial because of public insurance issues (Buselli et al., 2016).

According to the neurotrophic hypothesis, stress and depression are likely to be associated with a neurotrophins deficit leading to neuronal atrophy and cell loss in key limbic areas and in prefrontal cortex. Antidepressant treatments can block or reverse these effects (Banasr, Dwyer, & Duman, 2011; Sapolsky, 2001; Sheline, Gado, & Kraemer, 2003). A particular attention has been dedicated to Brain‐Derived Neurotrophic Factor (BDNF), a neurotrophin which plays a key role as allostatic mediator supporting functions such as memory and learning as well as continuous adaptations to environmental perturbations (Karatsoreos & McEwen, 2013). In chronic stress conditions, under the prolonged action of proinflammatory cytokines (TNF‐α, IL‐6, IL‐1β) and glucocorticoids, the BDNF gene is repressed, resulting in brain atrophy and contributing to the development of mental disorders in predisposed subjects (Anisman, 2009). Several recent studies demonstrated that peripheral (serum and/or plasma) BDNF levels are lower in patients suffering from mood disorders during manic/mixed and depressive episodes compared to matched healthy controls and that effective treatments are able to normalize them (Dell'Osso et al., 2010; Fernandes et al., 2011; Lin, 2009; Piccinni et al., 2008, 2009, 2015; Polyakova et al., 2015; Sen, Duman, & Sanacora, 2008). These data suggest peripheral BDNF levels as potential biomarkers of mood (depressive and manic/mixed) episodes, as well as predictors of treatments effectiveness.

On the other side, serum cortisol (seC) levels are a widely accepted index of energy mobilization and stress levels (McEwen, 1998). A meta‐analysis of cortisol awakening responses and psychosocial factors revealed that chronic psychological stress levels such as job stress and general life stress were associated with an increased cortisol awakening response (Chida & Steptoe, 2009). However, studies associating job stress with cortisol levels have yielded inconsistent results, with some studies reporting no association (Burton, Hinton, Neilson, & Beastall, 1996; Härenstam & Theorell, 1990), whereas others describing a link between low cortisol levels and high job stress (Steptoe et al., 1998).

The current literature is lacking regarding BDNF levels in patients suffering from AD or in workers exposed to occupational stress. Being AD a paradigm of stress‐related disorders, it seems very crucial to investigate about neuroplasticity markers such as peripheral BDNF levels and HPA‐axis functions in AD patients. The aim of this study was, therefore, to measure pBDNF and seC levels in workers exposed to occupational stress and suffering from AD compared to healthy control subjects and to investigate about possible correlations in AD patients between the biological variables and patients' clinical characteristics.

## 2 METHODS

### 2.1 Subjects

This study has a cross‐sectional design and examines two samples consisting, respectively, of 64 AD patients (33 M—51.6%, mean age ± *SD*: 47.3 ± 8.6 years) consecutively recruited at the Occupational Health Department operating in the Azienda Ospedaliero‐Universitaria Pisana and 38 healthy workers as control group (20 M—52.6%, mean age ± *SD *= 43.7: 10.5 years) recruited from blood donors in hospitals of Tuscany and Liguria.

The AD patients' group included cases of different clinical AD subtypes according to DSM‐IV‐TR classification (with depressed mood, with anxiety, with mixed anxiety and depressed mood, with disturbance of conduct, with mixed disturbance of emotions and conduct, unspecified). Exclusion criteria for patients were: age lower than 18 or higher than 65 years, presence of comorbid major neurological or medical illnesses, current acute or chronic inflammatory diseases in treatment with steroidal anti‐inflammatory drugs, taking psychopharmacological treatments in the last 4 weeks, pregnancy, presence of other current comorbid psychiatric disorders, presence of lifetime psychiatric comorbidity, onset of current AD symptoms as a reaction to preceding stressors different from current stressful work situations, insufficient comprehension of Italian that prevented completion of self‐report questionnaires, inability to sign informed consent.

Exclusion criteria for healthy controls were as follows: age lower than 18 or higher than 65 years, history of past and/or current major medical or mental disorders, heavy cigarette smoking, current treatment with steroidal anti‐inflammatory drugs, regular medication intake and/or substance abuse.

The research project was approved by the Ethics Committee of the University of Pisa according to the Declaration of Helsinki (2013) and the participation in the study was formalized with the collection of written informed consent.

### 2.2 Clinical assessment

The recruited AD patients received a general medical examination by an occupational physician and a psychiatric and psychological evaluation. The general medical examination, the collection of the medical history and the revision of clinical records of each patient allowed to exclude those with neurological or medical illnesses. Then, the patients were asked to complete questionnaires and rating scales as specified below.

A self‐report form was used to collect sociodemographic and work information, including the type of contract, the number of years of work in the current workplace and in previous workplaces, the size and the sector of the firm.

The clinical diagnosis of AD was confirmed by the administration of the Structured Clinical Interview for DSM‐IV axis I disorders Patient Version (SCID‐I/P; First, Spitzer, Gibbon, & Williams, 2002a) which also allowed, along with the psychiatric clinical evaluation, to exclude patients with other current comorbid psychiatric disorders or lifetime psychiatric comorbidity.

Work‐related stress was evaluated with the Job Content Questionnaire (JCQ), a widely used self‐administered workplace environment questionnaire designed to measure social and psychological characteristics of jobs (Karasek et al., 1998). Karasek's job strain model has been examined in relation to several different health outcomes and it has been used in several important studies, such as the Whitehall II Study (Marmot, Bosma, Hemingway Brunner, & Stansfeld, 1997). The JCQ best‐known scales, (a) decision latitude, (b) psychological job demands, and (c) social support, are used to measure the high‐demand/low‐control/low‐support model of job strain development. In particular, the decision latitude scale investigates the extent to which a worker can make decisions and exercise control over work (decision authority and skill discretion); the psychological job demand scale evaluates mental arousal or stimulation associated to the accomplishment of work tasks; the social support scale explores the amount of psychological and physical help at work given by supervisors/coworkers (Karasek et al., 1998).

Perceived psychological stress was measured by means of the Psychological Stress Measure (PSM), a validated self‐report instrument designed using 49 items drawn from descriptors generated by focus groups on stress (Di Nuovo, Rispoli, & Genta, 2000; Lemyre & Tessier, 1988).

The Beck Depression Inventory‐II (BDI‐II), a widely used 21‐item multiple‐choice self‐report test with two subscales (cognitive‐affective and somatic), was administered to AD patients to assess the severity of depressive symptoms (Beck, Steer, & Brown, 1996).

Anxiety and other psychopatological symptoms were evaluated by means of the Self‐rating Anxiety Scale (SAS; Zung, 1971) and the Self‐report Symptom Inventory (SCL‐90; Derogatis, Lipman, & Covi, 1973).

Sleep disturbances, which are frequent and early symptoms of work‐related stress, were studied by the administration of the Pittsburgh Sleep Quality Index (PSQI), a self‐report instrument with adequate skills of sensitivity, accuracy, and reproducibility (Buysse, Reynolds, Monk, Berman, & Kupfer, 1989).

Healthy workers were asked to complete the self‐report form for sociodemographic and work information, the PSM and the JCQ. Psychiatric diagnoses in controls were excluded by administering the Structured Clinical Interview for DSM‐IV axis I disorders Non‐patient Version (SCID‐I/NP; First, Spitzer, Gibbon, & Williams, 2002b). Medical comorbidity and substance abuse were also excluded in healthy controls by means of general medical examination, collection of the medical history and revision of clinical records.

### 2.3 BDNF and cortisol assays

To avoid a potential bias due to the presence of a diurnal rhythm of pBDNF and seC levels (Begliuomini et al., 2008), venous blood samples were drawn in the morning (between 8:00 and 10:00 a.m.). For plasma extraction, blood was collected into EDTA‐coated tubes that were kept on ice, centrifuged at 3,000 *g* for 10 min at 4°C and refrigerated at −80°C. The choice of EDTA as anticoagulant for pBDNF assay is consistent with the results of a study showing that heparin, but not EDTA, may interfere in some way with BDNF assay (Begliuomini et al., 2007). To measure the amount of total BDNF an enzyme‐linked immunosorbent assay (ELISA) was performed according to the manufacturer's kit instructions (Abcam's BDNF Human ELISA kit). Briefly, standards and samples were pipetted into a 96‐well plate and BDNF present in a sample was bound to the wells by the immobilized antibody specific for human BDNF. The wells were washed and biotinylated anti‐Human BDNF antibody was added. After washing away unbound biotinylated antibody, HRP‐conjugated streptavidin was pipetted to the wells. The wells were again washed, a TMB substrate solution was added to the wells and color developed in proportion to the amount of BDNF bound. The Stop Solution changed the color from blue to yellow, and the intensity of the color was measured at 450 nm.

For serum extraction, a tube of whole blood was collected and then left to clot at room temperature for 30 min. The clot was removed by centrifuging at 2,000 *g* for 10 min in a refrigerated centrifuge. Serum cortisol levels were assayed by the laboratory of Azienda Ospedaliero‐Universitaria Pisana using a commercial kit (Immunotech). Normal values for morning seC were 8.5–26 μg/dl.

### 2.4 Statistical analysis

The data were recorded in a specifically designed database and elaborated by means of the MedCalc software (version 12.7). According to the Kolmogorov–Smirnov test, all the examined continuous variables were normally (Gaussian) distributed except the PSM total score and the JCQ Job Demands and Social Support subscales scores. Therefore, the comparisons between patients' subgroups for variables of Gaussian distribution were performed by means of parametric statistical tests: in particular, the Student's test for independent samples was used. For the comparison of non‐Gaussian distributed variables the nonparametric statistical test of Mann–Whitney was performed. The Chi‐square test was used to compare the frequencies of categorical variables. Correlations between continuous variables of Gaussian distribution were examined by means of the Pearson's coefficient; for continuous non‐Gaussian distributed variables the Spearman's coefficient was used. A *p* value <0.05 was considered significant. As several correlation analyses were performed, the Benjamini–Hochberg correction for multiple testing was applied.

## 3 RESULTS

### 3.1 Comparisons between patients and controls

Sociodemographic and work characteristics, pBDNF and seC levels, stress rating scales scores of the two groups (AD patients and healthy controls) are reported in Table 1.

**Table 1:** Comparisons between AD patients and healthy controls (HC) for sociodemographic characteristics, stress rating scales scores, pBDNF, and seC levels

	AD patients (*n* = 64)	HC (*n* = 38)	*p* Value
Age (mean ± *SD*)	47.3 ± 8.6	43.7 ± 10.5	0.06
Gender, *M* (%)	33 (51.6%)	20 (52.6%)	0.9
Education ≥8 years, *N* (%)	63 (98.4%)	38 (100%)	0.8
Public company workers, *N* (%)	24 (37.5%)	10 (26.3%)	0.34
Private company workers, *N* (%)	40 (62.5%)	26 (68.4%)	0.7
Temporary job, *N* (%)	1 (1.6%)	6 (15.8%)	**0.02 (*χ*^2^ = 5.444)**
Stable job, *N* (%)	63 (98.4%)	30 (78.9%)	**0.003 (*χ*^2^ = 8.938)**
pBDNF (pg/ml; mean ± *SD*)	89.5 ± 53.2	41.0 ± 22.9	**<0.0001 (*t* = 5.316)**
seC (μg/dl; mean ± *SD*)	11.0 ± 4.3	11.3 ± 5.6	0.81
PSM total score (mean ± *SD*)	121.5 ± 23.2	70.0 ± 12.0	**<0.0001 *(Z *= 7.929)**
JCQ decision latitude (mean ± *SD*)	59.9 ± 14.7	66.5 ± 12.0	**0.02 (*t* = −2.334)**
JCQ job demands (mean ± *SD*)	36.1 ± 7.8	31.3 ± 7.4	**0.003 (*Z* = 2.946)**
JCQ social support (mean ± *SD*)	16.8 ± 5.0	23.8 ± 8.3	**<0.0001 (*Z *= 6.690)**

*Note*: *p*‐values and statistic values (*χ* ^2^, *t*, *Z*) for significant findings are reported in bold.

Abbreviations: AD, Adjustment Disorders; JCQ, Job Content Questionnaire; pBDNF, plasma Brain‐Derived Neurotrophic Factor; PSM, Psychological Stress Measure; seC, serum cortisol.

The two groups (AD patients and healthy controls) did not significantly differ in terms of age (*p* = 0.06), sex (*p* = 0.9) and education level having almost all patients (*n*. 63, 98.4%) and all controls at least 8 years of education (*p* = 0.8). The majority of patients (*n*. 40, 62.5%) and controls (*n*. 26, 68.4%) were working for private companies. The frequency of stable job was significantly higher in the patients' group than in the controls' one (98.4% vs. 78.9%, *χ* ^2^ = 8.938, *p* = 0.003). Plasma BDNF levels resulted significantly higher in patients than in controls (89.5 ± 53.2 pg/ml vs. 41.0 ± 22.9 pg/ml, *t* = 5.316, *p* < 0.0001), whereas no significant differences were found between the two groups for serum cortisol levels (*p* = 0.81). As for perceived psychological stress and occupational stress levels, patients presented significantly higher PSM and JCQ Job Demands scale but significantly lower JCQ Decision Latitude and Social Support scales scores than controls (see Table 1).

### 3.2 Correlation analyses

The results of correlation analyses between clinical and biological variables in AD patients are reported in Table 2.

**Table 2:** Matrix of correlation coefficients (*r*) between biological and clinical variables in AD patients' group (*n* = 64)

Variables	pBDNF levels	seC levels	PSM score	JCQ decision latitude score	JCQ job demands score	JCQ social support score	BDI‐II score	SAS score	SCL−90 score	PSQI score
pBDNF levels	1									
seC levels	**0.325** ***p* = 0.008**	1								
PSM score	−0.028	−0.180	1							
JCQ decision latitude score	0.094	−0.165	0.132	1						
JCQ job demands score	−0.115	−0.059	**0.288** ***p* = 0.02**	−0.007	1					
JCQ social support score	−0.144	−0.105	−0.030	0.119	−0.111	1				
BDI‐II score	−0.084	−0.130	**0.703** ***p* < 0.0001**	0.032	0.092	−0.221	1			
SAS score	−0.002	−0.120	**0.844** ***p* < 0.0001**	0.016	**0.290** ***p* = 0.02**	−0.007	**0.643** ***p* < 0.0001**	1		
SCL−90 score	−0.071	**−0.340** ***p* = 0.007**	**0.821** ***p* < 0.0001**	0.060	0.218	−0.137	**0.750** ***p* < 0.0001**	**0.760** ***p* < 0.0001**	1	
PSQI score	0.010	−0.218	**0.338** ***p* = 0.007**	−0.032	−0.056	−0.112	**0.353** ***p* = 0.007**	**0.384** ***p* = 0.002**	**0.329** ***p* = 0.01**	1

*Note*: *p*‐values and correlation coefficients (*r*) for significant findings are reported in bold.

Abbreviations: AD, Adjustment Disorders; BDI‐II, Beck Depression Inventory‐II; JCQ, Job Content Questionnaire; pBDNF, plasma Brain‐Derived Neurotrophic Factor; PSM, Psychological Stress Measure; PSQI, Pittsburgh Sleep Quality Index; SAS, Self‐rating Anxiety Scale; SCL‐90: Self‐report Symptom Inventory; seC, serum cortisol.

As regards biological variables, in AD patients but not in healthy controls pBDNF levels showed a statistically significant positive correlation with seC levels (*r* = 0.325, *p* = 0.008). In AD patients seC levels resulted to be negatively correlated only with the SCL‐90 total score (*r* = −0.340, *p* = 0.007). By extending correlation analyses to both AD patients and healthy controls, pBDNF levels were also found to be positively correlated with the PSM total score (*r* = 0.413, *p* < 0.0001) and negatively with the JCQ Social Support subscale score (*r* = −0.322, *p* = 0.001).

As for clinical variables, in AD patients the perceived psychological stress levels expressed by the PSM total score showed significant positive correlations with the total scores of many psychopathological rating scales such as the BDI‐II (*r* = 0.703, *p* < 0.0001), the SAS (*r* = 0.844, *p* < 0.0001), the SCL‐90 (*r* = 0.821, *p* < 0.0001) and the PSQI (*r* = 0.338, *p* = 0.007). In AD patients but not in healthy controls they were also positively correlated with the JCQ Job Demands subscale score (*r* = 0.288, *p* = 0.02).

Finally, in AD patients significant positive correlations were found between the SAS score and the JCQ Job Demands subscale score (*r* = 0.290, *p* = 0.02), the BDI‐II total score and the other psychopathological rating scales scores (SAS: *r* = 0.643, *p* < 0.0001; SCL‐90: *r* = 0.760, *p* < 0.0001; PSQI: *r* = 0.353, *p* = 0.007), the SAS total score and the SCL‐90 (*r* = 0.760, *p* < 0.0001) and PSQI (*r* = 0.384, *p* = 0.002) total scores, the SCL‐90 total score and the PSQI total score (*r* = 0.329, *p* = 0.01).

After adjusting by means of Benjamini–Hochberg correction for multiple testing, only two correlations lose statistical significance: (a) correlation between SAS score and JCQ Job Demands subscale score (*p* = 0.02 > BH value = 0.014) and (b) correlation between PSM total score and JCQ Job Demands subscale score (*p* = 0.02 > BH value = 0.015).

## 4 DISCUSSION, LIMITATIONS, AND CONCLUSIONS

As expected, the group of AD patients recruited at the Occupational Health Department operating in the Azienda Ospedaliero‐Universitaria Pisana showed higher levels of perceived psychological stress and occupational stress than healthy workers. In particular, on the basis of what emerged from the JCQ questionnaire, AD patients' jobs appeared to be characterized by greater job demands, lower social support and lower decision latitude compared to those of controls. Patients were more stressed despite having a more favorable working condition (almost all patients had a stable job) compared to controls.

In this study, AD patients' pBDNF levels resulted to be significantly higher than those of healthy workers, whereas no significant differences were found between the two groups for serum cortisol levels. To the best of our knowledge, there are not studies in literature investigating blood BDNF levels in patients suffering from AD. Conversely, it is largely demonstrated that peripheral blood (plasma and/or serum) BDNF levels are lower in patients suffering from mood disorders during manic/mixed and depressive episodes compared to matched healthy controls and that effective treatments are able to normalize them (Fernandes et al., 2011; Lin, 2009; Piccinni et al., 2008, 2009, 2015; Polyakova et al., 2015; Sen et al., 2008). A reduction of blood BDNF levels was also demonstrated in PTSD patients (Dell'Osso et al., 2009; Stratta et al., 2013) and in burnout patients (Onen Sertoz et al., 2008) compared with healthy controls. In particular, several recent studies focusing on HPA‐axis functions and brain imaging in patients suffering from burnout syndrome demonstrated that chronic occupational stress has a great impact in terms of HPA dysregulation, BDNF decrease, impaired neurogenesis, and limbic structures atrophy (for review see: Chow et al., 2018). Onen Sertoz et al. (2008) found no significant difference in terms of HPA‐axis function (basal seC, seC levels after 1 mg DST) but serum BDNF levels significantly lower in burnout patients than in healthy controls. Instead, in other disorders such as fibromyalgia (FM) both plasma and serum BDNF levels were reported to be higher in patients than in healthy controls, leading to hypothesize that BDNF increases in FM patients because involved in many compensatory modulatory mechanisms of pain (Haas, Portela, Böhmer, Oses, & Lara, 2010; Laske et al., 2007). Being AD by definition stress‐related disorders, it is possible that higher plasma BDNF levels in our AD patients than in controls are the expression of initial compensatory neuroprotective mechanisms from stress. Probably, when stress exposure overcomes these homeostatic mechanisms in susceptible individuals, this can cause the neurotrophins depletion and the neuroplasticity impairment up to brain atrophy which are reported in mood disorders, PTSD, and burnout cases (Chow et al., 2018; Dell'Osso et al., 2009; Pittenger & Duman, 2008). This hypothesis regarding initial BDNF levels elevation under stress exposure as homeostatic mechanism, is supported by some interesting preclinical evidence. For example, BDNF gene expression was reported to be increased in different rats' brain regions (hippocampus, amygdala, cortex) after different kinds of stress (maternal separation, social defeat, acute and chronic restraint; Bath, Schilit, & Lee, 2013), and an elevation of pBDNF levels was demonstrated in rats after both acute and chronic stress (Saruta et al., 2010; Tsukinoki et al., 2006).

Moreover, in our study AD patients' pBDNF levels were moderately and positively correlated with seC levels. On the contrary, in the study of Onen Sertoz et al. (2008) on burnout patients, there was no significant relationship between cortisol levels and serum BDNF levels. The positive correlation between seC and pBDNF levels found in this study could be interpreted as a part of the above hypothesized homeostatic neuroprotective mechanisms. In particular, BDNF could counteract the damaging effects of glucocorticoids excitotoxicity under stress. An interesting study by McMillan et al. (2004) demonstrated a significant increase in BDNF expression in macaques' hippocampus in response to chronic cortisol exposure: Authors hypothesized that such mechanism may protect brain against glucocorticoids excitotoxic insults. In healthy humans, Begliuomini et al. (2008) found a positive correlation between pBDNF and cortisol levels. Both BDNF and cortisol were significantly higher in the morning than in the night, showing a parallel diurnal rhythm. Such findings led Authors to speculate that these two factors may be physiologically co‐regulated, in order to maintain the homeostasis of integrated cerebral activities.

In our study, considering both AD patients and healthy workers, pBDNF levels also showed a moderate positive correlation with the levels of perceived psychological stress expressed by PSM total score and a moderate negative one with social support in the workplace (JCQ Social Support subscale). This result is in line with our hypothesis about the homeostatic and neuroprotective role of BDNF under stress but it is in contrast with other data from literature. In particular, a negative correlation between serum BDNF levels and work‐related psychological stress levels measured by means of Stress and Arousal Check List (s‐SACL) emerged in workers in health (Mitoma et al., 2008) and industrial settings (Okuno et al., 2011). However, these two studies included only healthy volunteers, whereas our sample is largely made by patients suffering from AD which resulted significantly more stressed than our healthy individuals. Therefore, such differences in the studied populations between the present research and the studies by Mitoma et al. (2008) and Okuno et al. (2011) could account for conflicting results.

A result difficult to discuss given the lack of similar findings in literature is the moderate negative correlation between seC levels and global psychopathology severity measured by means of SCL‐90. It could be possible that low seC levels characterize the most severe clinical pictures of trauma‐ and stressor‐related disorders as described in some PTSD patients (Doruk, Gulsun, & Balikci, 2015). A dampened response of the hypothalamic‐pituitary‐adrenal axis to environmental stressors could indeed negatively affect the activation of brain defence systems and cause more severe psychopathological manifestations (Yehuda & Seckl, 2011).

Finally, as expected we found in AD patients positive correlations of perceived psychological stress levels expressed by PSM total score with the scores of psychopathological rating scales: in particular the significant correlation was strong with depressive symptoms (BDI‐II), anxiety (SAS) and global psychopathology (SCL‐90), moderate with sleep disturbances (PSQI) and just weak with the levels of job demands detected by the JCQ. These findings are in line with many studies in literature reporting a significant association between the levels of perceived stress and symptoms of depression or anxiety in different populations (students, workers, patients; Bergdahl & Bergdahl, 2002; Ghorbani, Krauss, Watson, & Lebreton, 2008; Wiegner, Hange, Björkelund, & Ahlborg, 2015).

This study suffers from some limitations that should be acknowledged. First, the cross‐sectional design of the study and the relatively small sample size which did not allow further subgrouping, for example, for AD subtypes. Second, the psychiatric history as well as the occupational history were investigated and defined solely on the basis of what reported by the patients but no information from relatives was available. Another problem is related to the extent to which blood BDNF levels may reflect brain BDNF concentrations. However, since BDNF can cross the blood–brain barrier (Pan, Banks, Fasold, Bluth, & Kastin, 1998) and it has been observed that central and peripheral BDNF changes are positively correlated in rodents (Karege, Schwald, & Cisse, 2002), circulating BDNF is likely to contribute to protecting neural cells and maintaining their function. Moreover, we chose to investigate BDNF in plasma, because platelet‐poor pBDNF seems to be minimally affected by the amount of BDNF stored in platelets and, therefore, may represent a more reliable and sensitive marker of BDNF variations occurring in the brain and periphery (Lommatzsch et al., 2005). Finally, other factors known to influence blood cortisol and BDNF levels should be considered, such as the presence of a circadian rhythm with levels significantly higher in the morning (Begliuomini et al., 2008). To minimize this bias we chose to collect blood samples of patients and controls only once between 8:00 and 10:00 a.m. Despite these precautions, it is evident a discrepancy between our absolute BDNF values and some presented in recent publications which were found to be up to 100 times higher (Dell'Osso et al., 2010; Piccinni et al., 2008, 2009, 2015; Stratta et al., 2013). It is possible that by the acidification and neutralization of the samples (before ELISA protocols) according to the manufacturers' instructions for the used kits, in the above‐cited studies the total amount of BDNF was assayed, whereas our method may have measured only the amount of the free mature form. Therefore, we think that the different methodological procedure might explain the inconsistencies in the data reported in the literature.

In conclusion, this study demonstrates higher pBDNF levels in patients suffering from AD than in healthy controls and a positive correlation in AD patients between pBDNF levels and seC levels. As already mentioned the small sample size is an important bias of this study and affects the relevance of our findings which must be considered preliminary and need of validation in extended samples. However, the results of this study suggest that BDNF could play a key role in the pathophysiology of stress‐related disorders and that an elevation of its peripheral levels could contribute to protect neural cells under stress conditions. Since AD represent very common occupational stress diseases, research aimed at increasing the knowledge of their underlying neurobiological mechanisms and at investigating about the neuronal damage mediated by stress appears to be a real priority for preventive monitoring of workers exposed to occupational stress. Taking into account that work‐related stress represents an emerging risk in occupational medicine, the results of this study should therefore solicit further research focusing on putative biomarkers for stress‐related disorders as a potential tool for the diagnosis and prevention of occupational diseases.

## CONFLICT OF INTEREST

The authors report no conflicts of interest.

## Data Availability

The data that support the findings of this study are available from the corresponding author upon reasonable request.
